# Necrotizing fasciitis caused by *Stenotrophomonas maltophilia* on the neck of a previously healthy child^☆^

**DOI:** 10.1016/j.abd.2025.501179

**Published:** 2025-08-19

**Authors:** Giovanna Gelli Carrascoza, Denis Miyashiro, Murilo Perin da Silva, Talita Georgeana Baratieri Pinheiro, Suheyla Pollyana Pereira Ribeiro, José Antonio Sanches

**Affiliations:** a Dermatology Department, Faculty of Medicine, Universidade de São Paulo, São Paulo, SP, Brazil; b Pediatrics Department, Faculty of Medicine, Universidade de São Paulo, São Paulo, SP, Brazil; c Radiology Department, Faculty of Medicine, Universidade de São Paulo, São Paulo, SP, Brazil

Dear Editor,

Necrotizing fasciitis (NF) is a rare and potentially life-threatening soft tissue infection that spreads along the fascial planes, resulting in necrosis of the fascia and subcutaneous tissue.^1^ In adults, involvement of the head and neck structures is uncommon, with the lower extremities and trunk being the most prevalent locations. However, in children, the prevalence of lesions occurring in the head and neck is increased, reported to be 15% to 20%.^2^ We describe a case of a child who developed an extensive necrotic ulcer on the neck due to NF caused by *Stenotrophomonas maltophilia*.

A previously healthy two-year-old girl was admitted to the intensive care unit with fever, cough, and dyspnea for 15 days. She was diagnosed with acute respiratory failure, leading to orotracheal intubation. Five days later, she presented with swelling in the bilateral submandibular, submental, retroauricular, and occipital areas. Empirical broad-spectrum antibiotics (vancomycin + meropenem) were prescribed. Despite the antibiotic therapy, the lesions progressed, and the patient had a septic shock, prompting the patient’s transfer to a high-complexity hospital for further diagnostic evaluation. On her initial dermatological examination, she presented an extensive necrotic ulcer on the neck (Figs. 1A and B). A computed tomography scan (CT) revealed diffuse thickening and densification in the soft tissues of the neck (Fig. 1C). A punch biopsy and histopathological examination revealed coagulative necrosis in the epidermis, dermis, and hypodermis. In the fascia, there was a dense neutrophilic infiltrate with areas of necrosis (Fig. 2). Blood, tracheal, and skin cultures were obtained, all of which were positive for *Stenotrophomonas maltophilia* sensitive to levofloxacin and sulfamethoxazole/trimethoprim. The clinicopathological findings led to the diagnosis of necrotizing fasciitis.

**Figure 1:**
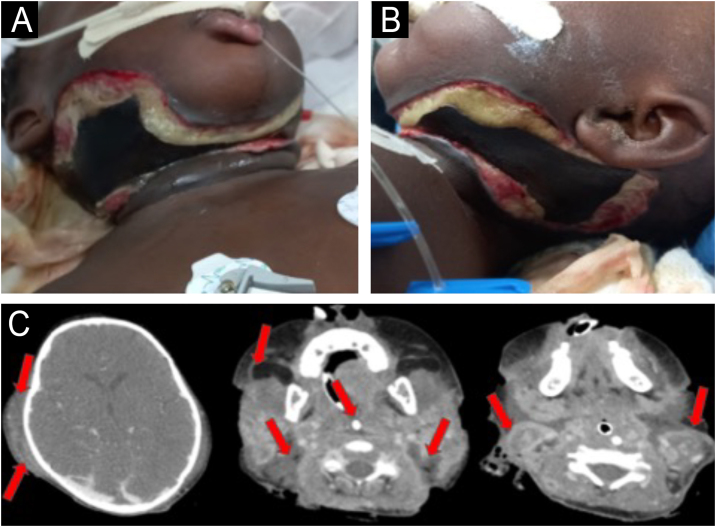
Extensive necrotic ulcer on the neck (A) right and anterior view (B) left view (C) CT scan showing thickening and densification of the myoadipose cervical planes and fascial enhancement (arrows).

**Figure 2:**
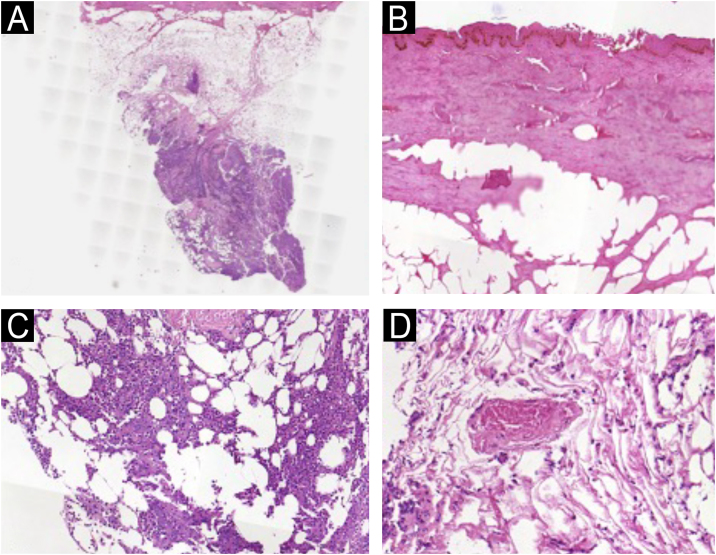
(A) Biopsy showing an intense inflammatory infiltrate in the fascia (Hematoxylin & eosin, ×20). (B) Epidermis with coagulative necrosis (Hematoxylin & eosin, ×20). (C) In the fascia, intense neutrophilic infiltrate, necrosis (Hematoxylin & eosin, ×20), and (D) thickening of vessels (Hematoxylin & eosin, ×20).

Based on the antibiogram results, adjustments to the antibiotic regimen were made with the incorporation of levofloxacin, and debridement was performed. Following clinical improvement, grafting was realized. The patient progressed without complications, eventually being discharged from the hospital without any lasting sequelae (Fig. 3).

**Figure 3:**
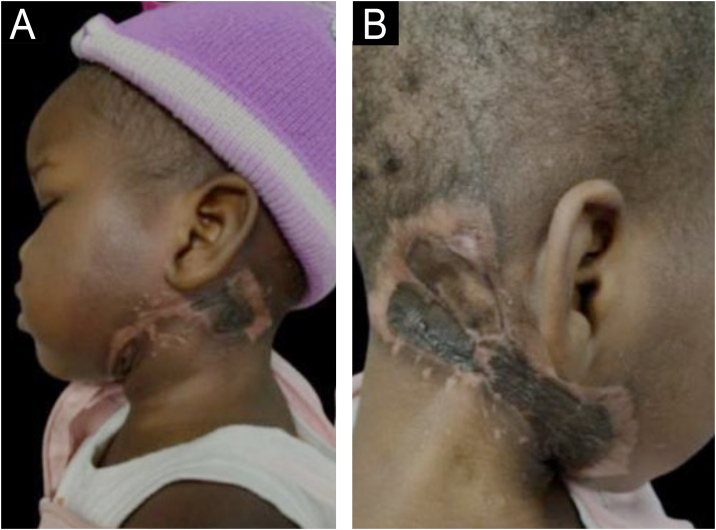
Patient after treatment.

The initial clinical presentation of NF is nonspecific, and the disease can be misdiagnosed as cellulitis, erysipelas, or abscess formation.^1^, ^2^ NF often initiates as a red, hot, and painful wound. As the disease advances, intense local pain transitions to numbness or analgesia.^1^ Progression of the pathological process involves necrosis of the subcutaneous and fascia, leading to arterial thrombosis, wet gangrene, and ultimately ischemic necrosis of the skin.^1^, ^3^ Inflammatory factors lead to fever, tachycardia, and eventually, septic shock.^1^, ^2^

In the pediatric population, NF exhibits distinctive features compared to adults. Although necrotizing fasciitis is often fatal in adults, its fatality rate seems to be lower in children.^4^ Unlike adult patients, where it is common in immunosuppressed and diabetic patients, children commonly affected are those previously healthy ones.^5^ Initiating features include minor and major trauma, surgical wounds, and varicella lesions.^2^ Also, unlike in adults, NF is usually a monomicrobial infection in children, most commonly owing to *Streptococcus pyogenes*, and is rarely polymicrobial.^4^

*Stenotrophomonas maltophilia* is a gram-negative aerobic bacillus that causes multi-drug-resistant opportunistic infections in hospitalized patients.^6^ These infections usually have high morbidity and mortality rates. The organism can form biofilms on various surfaces. Although the respiratory system is most commonly affected, *S. maltophilia* has also been reported to cause skin, soft tissue, and biliary tract infections, endophthalmitis, and meningitis.^6^ Risk factors include broad-spectrum antimicrobial therapy, prolonged stay in the intensive care unit, prolonged mechanical ventilation, immunosuppression, and the use of intravascular devices.^6^

The lack of distinctive lesions in the early stages of necrotizing fasciitis presents a significant diagnostic challenge, resulting in treatment delays. In our case, the delayed diagnosis led to septic shock and extensive tissue necrosis, indicative of advanced disease. When NF is suspected, it is imperative to obtain a contrast imaging exam (computed tomography or preferably magnetic resonance) to assess inflammatory changes, edema, fluid collection or abscesses, fascial enhancement, and gas formation.^1^, ^2^

Early and extensive surgical debridement is the mainstay of treatment. Immediate reconstruction is not recommended and should be delayed until the infection has been contained.^1^, ^2^, ^3^, ^4^, ^5^, ^6^ Cultures obtained from the tissue can guide long-term antibiotic therapy.

## Research data availability

Does not apply.

## Scientific Editor-in-Chief

Sílvio Alencar Marques.

## Financial support

The author(s) received no financial support for the research, authorship, and/or publication of this article.

## Authors’ contributions

Giovanna Gelli Carrascoza: Contributed to the approval of the final version of the manuscript, critical literature review, data collection, analysis and interpretation, intellectual participation in propaedeutic and/or therapeutic management of studied cases, manuscript critical review, preparation, and writing of the manuscript and study conception and planning.

Denis Miyashiro: Contributed to the approval of the final version of the manuscript, critical literature review, data collection, analysis and interpretation, effective participation in research orientation, intellectual participation in propaedeutic and/or therapeutic management of studied cases, manuscript critical review, preparation and writing of the manuscript and study conception and planning.

Murilo Perin da Silva: Contributed to the approval of the final version of the manuscript, data collection, analysis, and interpretation, intellectual participation in propaedeutic and/or therapeutic management of studied cases, and manuscript critical review.

Talita Georgeana Baratieri Pinheiro: Contributed to the approval of the final version of the manuscript, data collection, analysis, and interpretation, intellectual participation in propaedeutic and/or therapeutic management of studied cases, manuscript critical review.

Suheyla Pollyana Pereira Ribeiro: Contributed to the approval of the final version of the manuscript, critical literature review, data collection, analysis and interpretation, intellectual participation in propaedeutic and/or therapeutic management of studied cases, manuscript critical review, preparation and writing of the manuscript and study conception and planning.

José Antonio Sanches: Contributed to the approval of the final version of the manuscript, critical literature review, data collection, analysis and interpretation, effective participation in research orientation, intellectual participation in propaedeutic and/or therapeutic management of studied cases, manuscript critical review, preparation and writing of the manuscript and study conception and planning.

## Conflicts of interest

None declared.
